# Antioxidant Activity of Inulin and Its Role in the Prevention of Human Colonic Muscle Cell Impairment Induced by Lipopolysaccharide Mucosal Exposure

**DOI:** 10.1371/journal.pone.0098031

**Published:** 2014-05-16

**Authors:** Valentina Pasqualetti, Annamaria Altomare, Michele Pier Luca Guarino, Vittoria Locato, Silvia Cocca, Sara Cimini, Rossella Palma, Rossana Alloni, Laura De Gara, Michele Cicala

**Affiliations:** 1 Food Sciences and Human Nutrition Unit, University Campus Bio-Medico of Rome, Rome, Italy; 2 Gastroenterology Unit, University Campus Bio-Medico of Rome, Rome, Italy; 3 Surgery Unit, University Campus Bio-Medico of Rome, Rome, Italy; National Institute of Agronomic Research, France

## Abstract

**Background:**

Fructans, such as inulin, are dietary fibers which stimulate gastro-intestinal (GI) function acting as prebiotics. Lipopolysaccharide (LPS) impairs GI motility, through production of reactive oxygen species. The antioxidant activity of various fructans was tested and the protective effect of inulin on colonic smooth muscle cell (SMC) impairment, induced by exposure of human mucosa to LPS, was assessed in an *ex vivo* experimental model.

**Methods:**

The antioxidant capacity of fructans was measured in an *in vitro* system that simulates cooking and digestion processes. Human colonic mucosa and submucosa, obtained from disease-free margins of resected segments for cancer, were sealed between two chambers, with the mucosal side facing upwards with Krebs solution with or without purified LPS from a pathogenic strain of *Escherichia coli* (O111:B4) and inulin (Frutafit IQ), and the submucosal side facing downwards into Krebs solution. The solutions on the submucosal side were collected following mucosal exposure to Krebs in the absence (N-undernatant) or presence of LPS (LPS-undernatant) or LPS+inulin (LPS+INU-undernatant). Undernatants were tested for their antioxidant activity and the effects on SMCs contractility. Inulin protective effects on mucosa and submucosa layers were assessed measuring the protein oxidation level in the experimental conditions analyzed.

**Results:**

Antioxidant activity of inulin, which was significantly higher compared to simple sugars, remained unaltered despite cooking and digestion processes. Inulin protected the mucosal and submucosal layers against protein oxidation. Following exposure to LPS-undernatant, a significant decrease in maximal acetylcholine (Ach)-induced contraction was observed when compared to the contraction induced in cells incubated with the N-undernatant (4±1% vs 25±5% respectively, P<0.005) and this effect was completely prevented by pre-incubation of LPS with Inulin (35±5%).

**Conclusions:**

Inulin protects the human colon mucosa from LPS-induced damage and this effect appears to be related to the protective effect of inulin against LPS-induced oxidative stress.

## Introduction

Impairment of Gastro-intestinal (GI) motility is frequently observed during severe infection due to Gram-negative bacteria [1], [2]. One of the main virulence factors of these bacteria is the production of lipopolysaccharide (LPS), an endotoxin present in the bacterial cell wall, which is able to induce an immune/inflammatory host response. Several disorders, associated with intestinal motility disturbances and oxidative stress production, have been attributed to LPS [3]–[5].

This endotoxin is a potential mediator of multisystem organ failure and it has been shown that endotoxemia results in a significant impairment of intestinal smooth muscle contractility in animal models [6], by activating macrophages that may secrete several mediators including prostaglandins (PGs), H_2_O_2_, cytokines and nitric oxide; many of these mediators are also known to alter the kinetic properties of smooth muscle cells (SMCs) [7]. Results recently obtained have shown, in an experimental model, that exposure of the human colonic mucosa to pathogenic LPS affects muscle cell contractility and this effect is due to LPS translocation throughout the mucosal and submucosal layers, which leads to suppression of muscle cell contractility, via oxidative stress production [7]. Recent findings have revealed anti-inflammatory effects of Gram-positive probiotics, frequently used during acute GI infections, modulating the intestinal microbial flora balance [1]. In humans, the microbiota comprises a huge population of various bacterial species [8] and alterations in its composition result in reduced strength of the mucosal barrier, increasing translocation of GI pathogens into the systemic circulation of the host [9]. Interestingly, *Lactobacillus rhamnosus* GG (LGG), is able to counteract pro-inflammatory burst thus protecting human GI smooth muscle from pathogen LPS-induced damage [10], [11]. A recent investigation confirmed these data, showing that LGG prevents the morpho-functional modifications of colonic SMCs induced by LPS exposure, due to direct activation of Toll-like receptors (TLRs) expressed at the surface of human colonic SMCs and preventing the related production of pro-inflammatory cytokines [11].

Moreover, growing evidence indicates that also prebiotics, a category of nutritional compounds able to promote the growth of specific beneficial gut bacteria, represent an effective supporting treatment for several acute and chronic intestinal disorders [12]–[15]. Many dietary fibers, particularly soluble fibers, exhibit prebiotic activity [16], and amongst the various prebiotics, inulin and oligo-fructoses (also referred to as inulin-type prebiotics) have been the subject of extensive research [12]–[15]. However, despite the widespread use of fructans, for their prebiotic properties, and the increasing number of scientific reports regarding their beneficial effects [14], [15], [17]–[21], the exact mechanism by which they act remains to be defined. The potential mechanisms involved are: modulation of the immune system, prevention of the enterotoxin binding to the intestinal epithelium, regulation of intestinal microbial flora and direct effects on colonic motility [22]. Fructans reach the colon undigested, where they are then fermented by *Bifidobacterium spp*. and other lactic acid-producing bacteria. Potential fermentation products of inulin include short-chain fatty acids (SCFA) and butyrate, as a result of anaerobic fermentation [23]. These fermentation products have been demonstrated to be protective against several GI disorders, since they regulate, for example, colonic epithelial turnover and induce apoptosis in colon adenoma and cancer cell lines, acting at different stages of cancer onset [24]. Moreover, in human inflammatory bowel disease (IBD), in which an excessive immune response against gut microbiota has been demonstrated, the inulin-type fructans stimulate saccharolysis in the colonic lumen and favor the growth of indigenous lactobacilli and/or bifidobacteria. These effects are associated with reduced mucosal inflammation in experimental models of IBD [13].

Aim of the present study was to evaluate, in an experimental model, the possible protective effects of inulin against oxidative stress and colonic SMC contraction impairment, induced by exposure of human mucosa to LPS.

## Materials and Methods

The experimental protocols were approved by the Ethics Committee of Campus Bio-Medico University of Rome. Written Informed consent was obtained from all patients prior to surgery.

### Chemicals

Fructans with different botanical origin and different degree of polymerization (DP) and branching were analyzed; in particular, inulin from chicory roots (Sigma-Aldrich, Milan, Italy) linear with DP ranging between 10 and 30 [25]; inulin Frutafit IQ Instant Quality, (Sensus, Roosendal, The Netherlands) from chicory roots, linear with DP specifically of 8–13 monomers; inulin Frutafit TEX from chicory roots (Sensus, Roosendal, The Netherland) linear with DP specifically ≥22 monomers; agavins from *Blue agave* (Sensus, Roosendal, The Netherlands) branched molecules with non-certified DP presumably up to 30 monomers [26]–[28]; levans from *Bacillus subtilis* (Sensus, Roosendal, The Netherlands) linear with DP of approximately 125 [25]. In all cases, fructans were highly purified (90–99.5%) with a residual contamination of fructose, sucrose or glucose. All the other chemicals were purchased from Sigma-Aldrich, Milan, Italy.

### Antioxidant Capability Measurement and Fructan Determination

Antioxidant capability of fructans was assayed as scavenging activity toward 2,2′-azinobis-3-ethylbenzothiazoline-6-sulfonic acid (ABTS^•+^) monocationic radical according to the method described in the paper by Serpen *et al.* 2008 [29]. Total antioxidant capability was expressed as nmol of T*rolox* E*quivalent* A*ntioxidant* C*apability* (TEAC)/mg of sample (nmol TE mg^−1^).

Supernatants and undernatants (in contact with the mucosa and submucosa, respectively) and inulin subjected to simulated human digestion were also recovered and analyzed for their scavenging activity toward ABTS^•+^ radical with reference to the original method described in Re *et al.* 1999, slightly modified [30]. Supernatants and undernatants were filtered on Miracloth (Calbiochem, EMD Millipore, Billerica, MA, USA) under vacuum-seal in order to remove the potential presence of floating residuals of mucosa. During analysis, liquids were kept refrigerated opportunely. Inulin determination was performed on supernatants and undernatans using the AOAC 999.03 method. Megazyme’s Fructan kit (K-FRU 03/12, Megazyme International Ireland Ltd, Bray, Ireland) was used following the instructions provided by the supplier.

### In vitro Model of Digestion

The human digestive process of inulin was simulated in vitro by using the method described in Sayar *et al.,* 2005 [31]. Inulin Frutafit IQ solution (150 mg/mL) was subjected to a cooking step in boiling water for 4 minutes. After cooling to room temperature, the solution was progressively incubated with 50 mM pH 6.9 phosphate buffer for 15 minutes at 37°C. A sequence of incubations with various digestive enzyme solutions at specific pH values was carried out (see Table 1 for details). Briefly, the method was based on 3 sequential steps. The first consisted of initial incubation with human salivary α-amylase (EC 3.2.1.1; 5 mg/mL in 3.6 mM CaCl_2_) for 15 minutes at 37°C to simulate mouth conditions, followed by a second step of incubation at pH 2 with porcine pepsin (EC 3.4.23.1; 0.5 mg/mL in 0.9% NaCl) for 30 minutes at 37°C to simulate the gastric digestion. Finally, the third step of incubation at pH 6.9 was carried out adding pancreatin from porcine pancreas (0.5 mg/mL in Phosphate Buffer 50 mM pH 6.9) and bile acids standard mixture (containing 1.35 µM of sodium cholate, sodium deoxycholate, sodium glycocholate and sodium taurocholate) for 90 minutes at 37°C in order to simulate duodenal digestion. All incubations were performed in gentle agitation conditions (90 rpm) in an orbital shaker. The digested materials were recovered and analyzed for their total antioxidant capability.

**Table 1:** Steps of the cooking and digestion processes simulated in an *in vitro* system.

TREATMENTS	NOT TREATED	COOKED	DIGESTED	COOKED and DIGESTED
Boiling 4′ and cool to room temperature	−	+	−	+
Add 10 mL Phosphate Buffer 50 mM pH 6.9	−	−	+	+
Stir slowly (90 rpm) 15 min 37°C	+	+	+	+
Add 38.5 µL of human salivary α-amylase (**MOUTH COMPARTMENT**)	−	−	+	+
Stir slowly (90 rpm) 15 min 37°C	+	+	+	+
Adjust pH to 2.0 with HCl (**STOMACH COMPARTMENT**)	−	−	+	+
Add 96.2 µL of porcine pepsin	−	−	+	+
Stir slowly (90 rpm) 30 min 37°C	+	+	+	+
Adjust pH to 6.9 with NaOH (**SMALL INTESTIN COMPARTMENT**)	−	−	+	+
Add 192.3 µL of pancreatin from porcin pancreas	−	−	+	+
Add 3.1 mL of bile acids standards mixture	−	−	+	+
Stir slowly (90 rpm) 90 min 37°C	+	+	+	+

The human digestive process of inulin was simulated *in vitro* by using the method described in Sayar et al. 2005 (31).

### Tissue Specimens

Normal colonic mucosa was obtained from the healthy margins of cancer resections from six patients with adenocarcinoma of the colon (M:F = 4∶2, age range 48–73 years), treated at the Campus Bio-Medico University of Rome (Italy) between September 2010 and November 2012. None of these patients had a history of colonic motility or of a neuromuscular or collagen disorder; specimens with *diverticula* were excluded. Hemi-colectomy was performed in all patients and a specimen from the resected colon was obtained, at a distance from the area involved by the carcinoma. Fresh specimens were brought to the laboratory in oxygenated (95% O_2_ and 5% CO_2_), chilled Krebs solution containing (in mM) 116.6 NaCl, 21.9 NaHCO_3_, 1.2 KH_2_PO_4_, 5.4 glucose, 1.2 MgCl_2_, 3.4 KCl, and 2.5 CaCl_2_.

### Experimental Set-up

After removal of the muscle layer and serosa, the tissue containing mucosa and submucosa was sealed between two tubes with the luminal side of the mucosa facing upwards and submucosal side facing downwards, as previously described [32]. Inflammation conditions were induced by means of LPS from *Escherichia coli* pathogenic strain serotype O111:B4 (Sigma-Aldrich, Milan, Italy) exposure.

**Figure 1:**
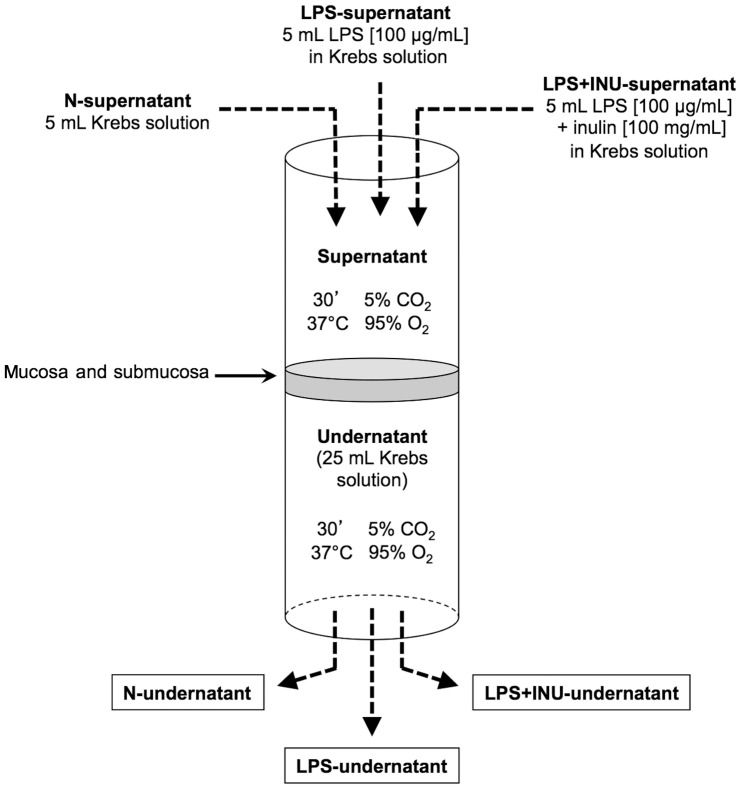
Schematic representation of the experimental set-up.

In the experimental model the luminal side of the mucosa was overlayed with 5 ml of Krebs (N-supernatant), 5 mL of 100 µg/mL LPS solution in Krebs (LPS-supernatant), or with 5 mL of LPS +100 mg/mL inulin Fructafit IQ solution in Krebs (LPS+INU-supernatant). Whole the biological system was maintained for 30 minutes at 37°C in a thermostatic bath, which was constantly oxygenated. Smaller and larger tubes were adopted to guarantee that the set-up was well sealed; the efficacy of this operation was ensured by monitoring the level of the solution in the smaller tube (Figure 1).

After 30 minutes, the Krebs solutions on the submucosal side, in the absence (N-undernatant) or presence of LPS (LPS-undernatant) and LPS-Inulin (LPS+INU-undernatant), were collected to evaluate their effects on SMC contractility.

### Measurement of Protein Oxidation

Protein oxidation was evaluated in the colonic mucosa by measuring carbonyl group content assayed using the dinitrophenylhydrazine [33]. Carbonyl content was calculated using an extinction coefficient of 22000 M^−1^ cm^−1^ [33].

### Ex vivo Experiments on Human Colonic Smooth Muscle Cell (SMC) Motility Isolation of SMCs

Colonic smooth muscle tissue was separated from the submucosa by sharp dissection in a bowl containing Krebs solution in adequate oxygenation conditions (95% O_2_ and 5% CO_2_) to minimize stress induced on the tissue. Macroscopic blood vessels of muscle tissue were carefully removed; the remaining slices of the circular muscle layer were then cut into small strips (∼1 mm wide) and the muscle cells were isolated by means of enzymatic digestion as described elsewhere (34) in order to obtain pure and homogeneous primary human colonic smooth muscle cells, devoid of any neuro-immune contamination. Briefly, HEPES buffer contained 10 mM HEPES, 1 mM CaCl_2_, 4 mM KCl, 125 mM NaCl and 1 mM MgCl_2_. Upon analysis, 10 mM of glucose were added, then 20 mL of this solution were added with 0.25 mM ethylene diamine tetra acetic acid (EDTA), 1 mg/mL bovine serum albumin (BSA) and 1 mg/mL papain from *Papaia latex*. The solution was adjusted to pH 7.2 and 0.5 mg/mL collagenase type F from *Clostridium histolyticum* was added. The tissue was maintained in an enzyme solution at 4°C for ∼3 hours, kept at room temperature for 30 minutes, then incubated in a water bath at 31°C for ∼30 minutes to allow the release of single smooth muscle cells (SMCs). At the end of enzymatic digestion period, single SMCs were isolated pouring the suspension on a 200 µm Nitex mesh (Tetko Elmsford, NY, USA). The remaining SMCs were rinsed with collagenase-free HEPES-buffered solution to remove any possible trace of collagenase (therefore approximately three-four times with 3–4 mL of solution). Thereafter, the rinsed SMCs were transferred inside clean beakers containing fresh HEPES-buffered solution (usually 1 mL for every sample to be analyzed) and these were mixed together to obtain a homogeneous cell suspension. All reagents used for the preparation of solutions were purchased from Sigma-Aldrich, Milan, Italy.

### Assessment of Biological Activity of SMCs

Isolated colonic SMCs were analyzed to assess their biological activity. For this purpose, SMC suspension was mixed with the undernatants collected in the set-up, as described herewith, in 1∶1 ratio and pre-incubated for 30 minutes at 37°C in agitation and oxygenation conditions.

At the end of the pre-incubation period, to assess the biological activity of SMCs, the cells from each group were exposed to a maximum dose of Ach (10 µM) for 30 seconds and, thereafter, fixed in acrolein at a 1% final concentration and refrigerated. In order to measure cell length, a drop of the cell-containing medium was placed on a glass slide and 30 consecutive cells from each slide were observed through a phase-contrast microscope (model Eclipse E400; Nikon Instech Co., Kawasaki, Japan). A closed-circuit television (TV) camera (Nikon Instech Co., Kawasaki, Japan) connected to a PowerPC computer (Hewlett-Packard Company, Palo Alto, CA, USA) was used to obtain images. Cell lengths were measured using a computer software programme (Image 1.33; National Institutes of Health, Bethesda, MD, USA).

The mean length of 30 cells measured in the absence of agonists was assumed as the control length and compared with lengths measured following the addition of test agents. Shortening was defined as percent decrease in average length following agonist treatment, compared with the control length.

Images of colon muscle cells were obtained by means of a phase contrast optical microscope (Leitz DMRB Leica model) at 40× magnification using the NIH Elements FreeWare imaging software program 2.10 version and closed-circuit television camera (Nikon Digital Sight DS-U1) connected to a computer.

### Statistical Analysis

Statistical analysis were performed on all experimental results by GraphPad Prism statistical software program 4.02 version. One-way analysis of variance (ANOVA) and non-parametric tests were used with P values determined using Tukey’s comparison test. Differences were considered to be statistically significant with P<0.05. Data of cells contraction are expressed as mean ± standard error (SE) of 6 individual experiments. The average length of 30 cells measured in the absence of agonists was assumed as the control length and compared with lengths measured after the addition of test agents. Student’s *t* test was used for statistical analysis. A P value <0.05 was considered significant.

## Results

### Antioxidant Properties of Fructans

**Figure 2:**
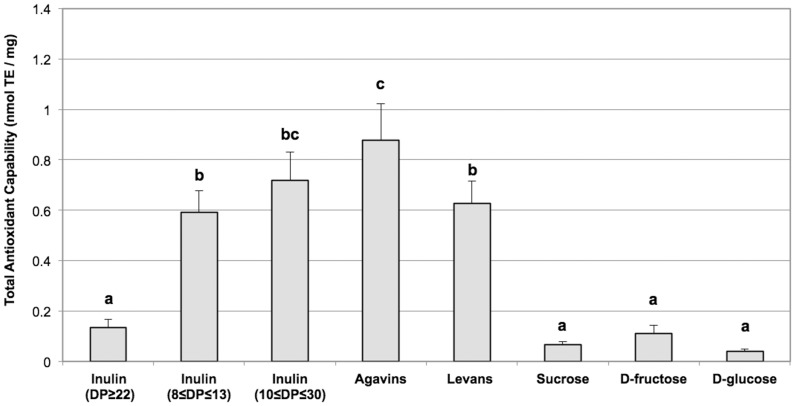
Total Antioxidant Capability of fructans. Fructans, with different molecular characteristics (DP and branching) and botanical origins, were tested for their antioxidant capability and compared to glucose, fructose and sucrose. Reported values are the means of six independent experiments ± SE. Values marked by the same letter are not statistically different (P>0.05; ANOVA test); values marked by different letters are statistically different (P<0.05; ANOVA test).

**Figure 3:**
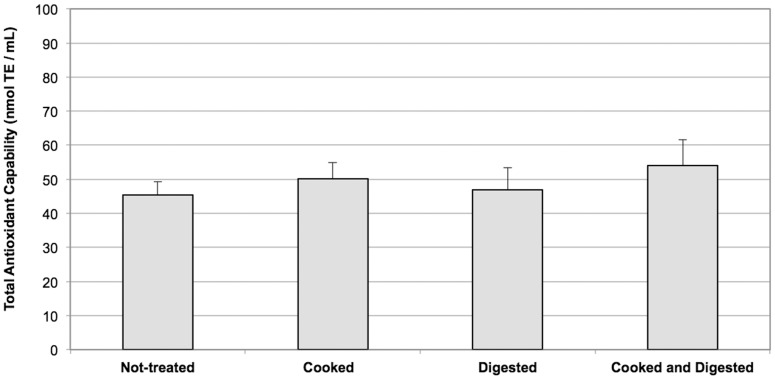
Effects of cooking and digestion on total antioxidant capability of inulin. Inulin (150 mg/ml) was subjected to cooking and digestion processes by an *in vitro* system as described in Material and Methods. Reported values are the means of six independent experiments ± SE.

Total antioxidant capability of fructans, with different degrees of polymerization (DP – 8–125) and molecular structures (linear or branched), was analysed by the TEAC method and compared with the antioxidant activity of simple sugars forming fructan polymers (glucose, fructose and sucrose). All the fructans tested, with the exception of inulin Frutafit TEX, showed greater antioxidant capability than sucrose, glucose and fructose. Indeed, fructans from *Blue agav*e and inulin obtained by means of Sigma from chicory roots appeared to have the greatest antioxidant capability, even if the values obtained from inulin Fructafit IQ and Levans were not much lower (Figure 2). The different fructans had variable solubility in water, with inulin Fructafit IQ showing the highest solubility (up to 200 mg/ml) and inulin Frutafit TEX the lowest (up to 5 mg/ml). The antioxidant capability of the analysed fructans showed a linear dose-dependent trend in the range of their solubility (data not shown). On account of the high solubility and good antioxidant capability and since it had already been commercially adopted as a food additive, inulin Fructafit IQ was selected for the experiments described herewith. As shown in Figure 3, the antioxidant activity of inulin Fructafit IQ was not affected by treatments at high temperature thus simulating cooking processes, as well as by pH changes and exposure to digestive enzymes typically occurring during the food transit through the GI tract.

### Fructan Protection against LPS- induced Damage in Colon Tissue

**Figure 4:**
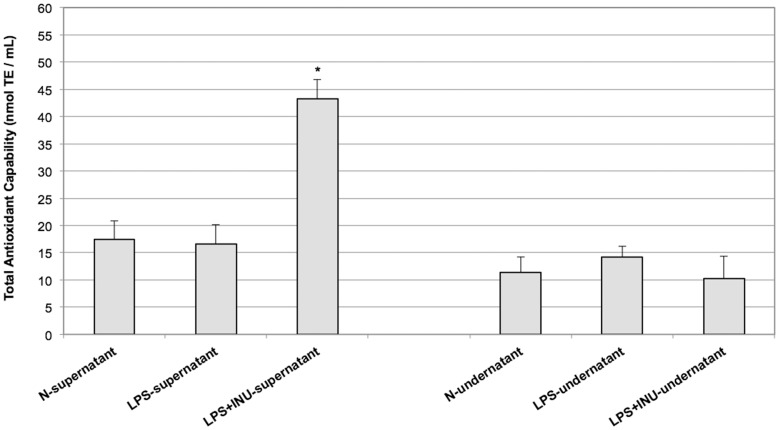
Total antioxidant capability of N, LPS and LPS+INU-supernatants. The supernatants were collected after 30 minute of mucosal exposure and analysed. Reported values are the means of six independent experiments ± SE. (*P<0.001; ANOVA test).

In order to investigate the putative protective effect of inulin on the human colon following injury induced by pathogenic bacteria, *ex vivo* experiments have been performed. Colonic mucosa and submucosal layers were mounted in three chambers with mucosa exposed to Krebs solution (N-supernatant), Krebs solution containing LPS (LPS-supernatant) and Krebs solution containing LPS plus inulin (LPS+INU-supernatant). The submucosa was in contact with Krebs solution in all the chambers (N, LPS and LPS+INU-undernatants - see Material and Methods for details). After 30 minutes of treatment total antioxidant capability of the supernatants and undernatants were measured. LPS+INU-supernatant had the highest antioxidant capability, while no differences were found between N- and LPS-supernatants and in any of the undernatants collected in the three experimental conditions (Figure 4). In order to ensure that inulin did not cross the colonic mucosa and submucosa layers, the presence of inulin was investigated in the LPS+INU-undernatant. The data obtained confirmed that inulin cannot pass through the mucosal and submucosal layers and thus cannot move from the supernatant to the undernatant thus further validating the integrity of the colon tissues used in the experiments described herewith (data not shown).

**Figure 5:**
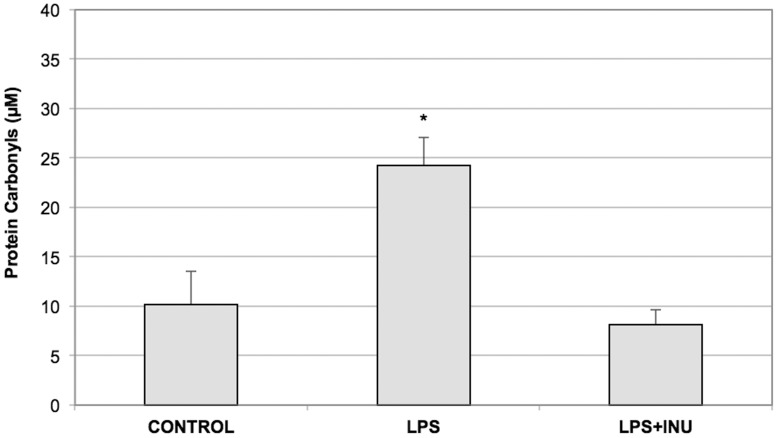
Levels of protein oxidation in colonic mucosa and submucosa layers following the exposure to N, LPS and LPS+INU-supernatants. Protein oxidation level was measured in all the analysed experimental conditions as total protein carbonyl group content. Reported values are the means of six independent experiments ± SE. (*P<0.01; ANOVA test).

Since by using the same experimental system it has been demonstrated that LPS exposure is responsible for oxidative stress on the colonic mucosa [20], the protective effect of inulin against oxidative damage was investigated in the colon tissue exposed to LPS. For this reason, the level of the carbonyl groups, a marker of protein oxidation, was evaluated in the colon tissues subjected to the three experimental conditions. The thirty-minute exposure of the mucosa to LPS was responsible for inducing a significant increase in the carbonyl groups of the colonic mucosa proteins compared to the control tissues, thus confirming the presence of the LPS-induced oxidative stress that determines protein oxidation. Interestingly, when the colonic mucosa was exposed to the LPS+INU-undernatant, the amount of the carbonyl groups was maintained almost identical to that of controls (Figure 5).

### Effects of N-undernatant, LPS-undernatant and LPS+INU Undernatant on Muscle Cell Contractility

**Figure 6:**
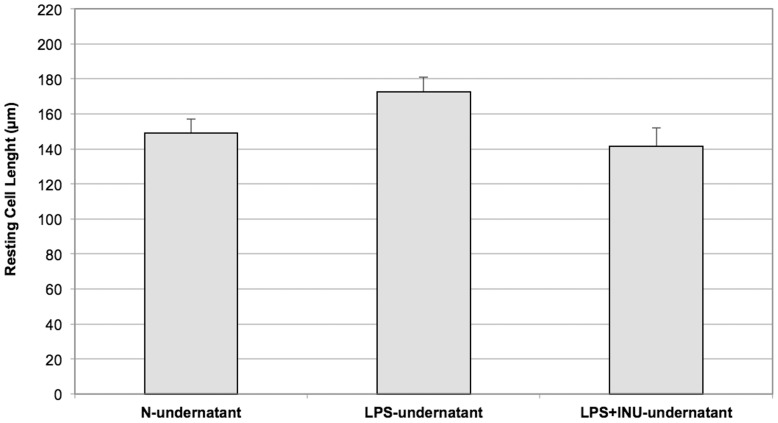
Effect of the exposure to N, LPS and LPS+INU-undernatants on resting length of colonic smooth muscle cells (SMCs). Cell length was measured in the absence of agonists. Reported values are the means of six independent experiments ± SE.

**Figure 7:**
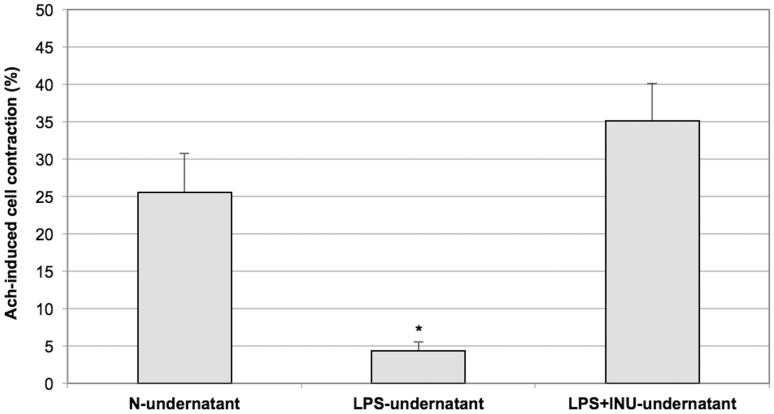
Effect of the exposure to N, LPS and LPS+INU-undernatants on Ach-induced contraction of colonic smooth muscle cells (SMCs). The average length of 30 cells measured in the absence of agonists was assumed as the control length. Reported values are the means of six independent experiments ± SE (*P<0.005; Student’s t test).

Pre-incubation with the N-undernatant did not induce morphological changes in SMCs. The thirty-minute exposure to the LPS-undernatant and to the LPS+INU-undernatant did not decrease cell length compared to SMCs exposed to the N-undernatant (172.5±8.5 and 141.5±10.4, respectively, vs 149.05±8.1 µm, P = ns. Figure 6). Following thirty-minute exposure to the LPS-undernatant, a significant decrease in maximal Ach-induced contraction was observed when compared to the contraction induced in cells incubated with the N-undernatant (4±1% vs 25±5% respectively, P<0.005. Figure 7) and this was completely prevented by pre-incubation of the LPS with Inulin (35±5%, P = ns versus N-undernatant, Figure 7).

## Discussion

Data emerging from the present investigation demonstrated that pre-treatment with inulin prevents impairment of the muscle cell contractility induced by LPS exposure, possibly counteracting the mucosal production of free radicals.

It is well known that LPS reduces intestinal smooth muscle contractility at various levels of the GI tract in animal models [35]–[39]. Moreover, we have recently demonstrated, employing the same experimental model used in this study, that acute exposure of normal human colonic mucosa to pathogenic LPS impairs muscle cell contractility [7]. It has been suggested that this effect is due to excessive mucosal oxidative stress and, partially, to LPS translocation through the luminal layers: both factors would lead to an increase in the intra-cellular production of inflammatory mediators via nuclear factor-kappaB (NF-kB) activation [7]. These data add further evidence, together with findings emerging from previous studies [40]–[42], to the pivotal role of oxidative stress in the pathogenesis of endotoxin-induced motility disorders during sepsis. Moreover, in animal models of colitis and in patients with ulcerative colitis, the release of free-radicals from the intestinal mucosa would appear to trigger the inflammatory response which affects the deeper layers [40].

It has recently been hypothesized, in *in vitro* studies, that fructans have reactive oxygen species (ROS) scavenging capability [43], thus supporting their beneficial effects during intestinal inflammatory processes [44]. The present results confirmed the significant antioxidant activity of inulin (Figure 2). Indeed, this antioxidant capability is much higher than that of fructose, glucose and sucrose, all of which are constituents of fructans. A putative role of oligofructoses, in counteracting the pro-oxidative effects of a high fructose diet, has been demonstrated in rats, where the addition of fructans to the diet has been suggested to provide an early defense against oxidative stress, starting before the activation of the endogenous ROS detoxification systems [45]. Moreover, *in vivo* ROS scavenging capability of certain sugars has also been supported by the evidence that intra-peritoneal administration of synthetic oligosaccharides determines a dose-dependent decrease in lipid peroxidation [46].

Interestingly, inulin would appear to be able to contrast the oxidative damage induced by LPS in the colonic tissue since the level of protein oxidation induced by LPS exposure was remarkably reduced when the tissue was treated with LPS plus inulin (Figure 5). These results are in keeping with our previous data which indicated that LPS induces oxidative stress in the colonic tissue [7]. Moreover, these data confirm that inulin can protect the colonic mucosa, not only by means of its positive effect as prebiotic on the microbiota, but also acting as a barrier against oxidative stress, consistently with the recent suggestion of its putative ROS scavenging capability (Figure 2) [45], [47]. In order to explore the protective role of inulin, the antioxidant capability was assessed in the supernatants and undernatants collected in all the experimental conditions analyzed. As expected, the antioxidant capability was increased only in the supernatant in which inulin has been added (LPS+INU-supernatant). Conversely, no difference in the antioxidant capability of the undernatants derived from all the tested experimental conditions, was observed (Figure 4), thus confirming that inulin does not pass through the mucosal and submucosal layers, but exerts its protective role on the mucosal side. The finding that the presence of inulin in the LPS+INU-undernatant blocks the cascade events, triggered by LPS, is also confirmed by the different effects of the undernatants collected in control, LPS- and LPS+INU conditions on muscle contractility.

To confirm that the scavenging activity of inulin is preserved following cooking and digestion processes, the antioxidant capability of several fructans was tested by means of thermic treatments, that mimic cooking, as well as by treatments simulating the changes in pH and the exposure to lytic enzymes occurring during digestion (Figure 3). Following both of these procedures, the antioxidant capability remains unchanged. This is of particular interest since many other soluble antioxidants, consumed during diet, lose their ROS scavenging capability during cooking and digestion. Vitamin C, for example, loses between 40–90% of its antioxidant properties when subjected to high temperature [48], [49]. Fructans, resistant to thermic treatment and digestion, increase their role as antioxidant molecules also since the activity of these can be performed in the colon, a GI tract that is not reached by other ROS scavengers, such as vitamins C and E, which are absorbed in the first part of human gut [50]–[52].

In conclusion, these results, indicating the antioxidant effect of inulin, add further evidence to the prevention of the impairment of muscle cell contractility induced by mucosal exposure to LPS and pave the way for further investigations assessing the role of inulin in the treatment of post-infectious irritable bowel syndrome (IBS), as well as in the prevention and treatment of inflammatory diseases related to oxidative stress. Further studies are also necessary to better understand whether the beneficial effects of inulin on the health of the host are attributable, not only to its bifidogenic action, but also to its antioxidant properties.
